# Editorial Notes

**Published:** 1927

**Authors:** 


### Editorial Notes.

**Centenary of Lord Lister's birth, April 5th, 1927.**

THE twentieth century is apt to look back on the nineteenth with scornful pity. Gentlemen with their dusters have been busy amongst the figures that were thought grand in pre-war days. But the most vigorous post-war dusting only enhances the reputation of Lister. His pre-eminence in surgery stands unchallenged. Born five years after Pasteur, he divides with him the credit for the whole of the progress made in medical science during the latter half of the nineteenth century. His acknowledgment to Pasteur was unstinted: "The philosophical investigations of Pasteur long since made me a convert to the Germ Theory, and it was on the basis of that theory that I founded the antiseptic treatment of wounds in Surgery." Lister's discovery was not made by chance. As we read to-day of his untiring energy in devising experiments to prove his thesis we are amazed at the fertility of his brain. As we read of the small impression his scientific proofs made on his surgical contemporaries we stand aghast at the mentality of the men to whom for the most part the surgery of his day was entrusted. Fortunately for the welfare of humanity Lister had the gift of making disciples among his students, and although his contemporaries were antagonistic, and sometimes virulently so, to his new teaching, he entered as a candidate for the Professorship of Surgery in Edinburgh supported by an invitation signed by over a hundred students of that University. Lister lived long enough to see his principles adopted by the whole civilised world, and when he died in 1912 at the age of eighty-five he had been the recipient of honours and distinctions from every part of the world. There has lately been some controversy over the decision of the managers of the Glasgow Royal Infirmary to pull down the old ward where Lister initiated and almost perfected his methods of antiseptic surgery.[1] The question is settled now, the ward has gone from Glasgow, but part of it has been rescued and reconstructed in the Wellcome Historical Medical Museum in London.[2] Here it will take its rightful place among the relics of workers and discoverers in Medicine to whom posterity will delight to pay due honour. For the *mot juste* to express the debt of humanity to Joseph Lister we can turn to the inscription placed on a brick taken from the Lister Ward in Glasgow to the University of Rochester: "His work continues here and everywhere outlasting brick and stone." This thought seems almost to justify the splendid insolence of the Infirmary managers in destroying Lister's ward.

Our readers will welcome the personal reminiscences of Lord Lister which are embodied in Mr. Richardson Cross's article on p. 96 of this issue.

**The Colston Research Society.**

THE Annual Dinner of the Colston Research Society was held at the Victoria Rooms, Clifton, on Monday, May 23rd, under the Presidency of Dr. W. W. Ward, and was well attended. The guests of the evening were Sir William

[1] *Lister and the Lister Ward; a Centenary Contribution.* Glasgow: Jackson, Wylie & Co. 1927. Price 12/6.

[2] *Lister Centenary Exhibition Handbook.* London: The Wellcome Foundation Ltd. 1927.

Hardy and Sir Humphry Rolleston, Bart., whose speeches, in response to the toast of their health, proposed by Sir Lionel Goodenough Taylor (Master of the Society of Merchant Venturers), were listened to with much appreciation. The collection was announced at £764 1s. 0d., of which £200 has been ear-marked by the donors for a Colston Research Fellowship in the Institute of Cardiac Research, to be established by Dr. Carey Coombs at the General Hospital. The Institute will be equipped by the University, and the Colston Research Fellowship will be available for the purposes of a skilled assistant to work under the supervision of Dr. Coombs. Owing to the generosity of the donors, this Fellowship will be tenable for a period of two years at £200 a year.

The collection, by comparison with recent years, was satisfactory (apart from the special donation), but the sum collected annually is not large in consideration of the many important claims made upon it, and in view of the opportunities which the University now offers it is to be hoped that the list of subscribers may continue to grow so that the amount collected annually may be in the region of four figures.

**The Geographical Distribution of Rheumatic Heart Disease.**

ONE of the principal tasks to be undertaken by the Institute of Cardiac Research is the collection of records of cases of rheumatic heart disease arising during childhood in the area comprised within the counties of Gloucestershire, Somerset and Wilts. The purpose of this inquiry is to ascertain the geographical and other conditions that predispose to this disease. The clerical expenses are undertaken by the Medical Research Council and other public bodies are contributing assistance. We understand that the practitioners of the area in question will also be asked to help by furnishing records of cases arising within their observation, and we venture to appeal to them to do all that they can to ensure that the project shall be successful.

---

### Meetings of Societies.

